# Intractable Pleural Effusion After Stereotactic Ablative Radiotherapy for Early-Stage Lung Cancer

**DOI:** 10.7759/cureus.36925

**Published:** 2023-03-30

**Authors:** Atsuto Katano, Masanari Minamitani, Yuki Nozawa, Hideomi Yamashita, Keiichi Nakagawa

**Affiliations:** 1 Department of Radiology, The University of Tokyo Hospital, Tokyo, JPN; 2 Department of Comprehensive Radiation Oncology, Graduate School of Medicine, The University of Tokyo, Tokyo, JPN

**Keywords:** adverse event, early-stage lung cancer, pleural effusion, complication, stereotactic ablative radiotherapy

## Abstract

Stereotactic ablative radiotherapy (SABR) is an effective and attractive treatment option for patients who are poor surgical candidates. This case report describes a rare but serious complication of intractable pleural effusion after SABR for early-stage lung cancer. The patient was an 89-year-old woman with a medical history of early-stage breast cancer who was treated with partial resection and postoperative radiotherapy of 50 gray (Gy) in 25 fractions. SABR using 55 Gy in four fractions was conducted for lung lesions. The patient developed a pleural effusion that was refractory to conservative management and required multiple interventions, including repeated thoracentesis. This case report emphasizes the importance of monitoring and managing pleural effusion in patients with lung cancer receiving radiotherapy.

## Introduction

Stereotactic ablative radiotherapy (SABR) is a unique technique that utilizes irradiation precisely targeting the tumor while minimizing radiation exposure to adjacent normal tissue. With this therapy, tumors of small to moderate size can be treated with a single or a limited number of doses of radiation. According to the revised STARS trial, for early-stage non-small cell lung cancer, SABR presented an overall survival rate comparable to that of the surgical approach in a propensity-matched analysis (hazard ratio = 0.86; 95% CI = 0.45-1.65, p = 0.65) [1]. Moreover, SABR was associated with lower risks of respiratory adverse events at three months after radiotherapy (odds ratio = 0.51; 95% CI = 0.31-0.86) compared to surgical limited resection [2]. However, the risk of adverse events should be carefully monitored. Timmerman et al. reported that the incidence rate of SABR-related grade 3 adverse events was 12.7% and that of grade 4 adverse events was 3.6% [3].

Radiation-induced pleural effusion is a serious complication of thoracic radiotherapy. Although the exact mechanism of pleural effusion is unknown, progressive pulmonary fibrosis, abnormal vascular permeability, and lymphatic obstruction are possible contributing factors to the accumulation of exudate fluid in the pleural space [4]. Pleural effusion after thoracic radiotherapy occurs in the first six months, and a small volume persists for several months [5]. We report an intractable pleural effusion after SABR, which persisted for more than two years.

## Case presentation

**Figure 1:**
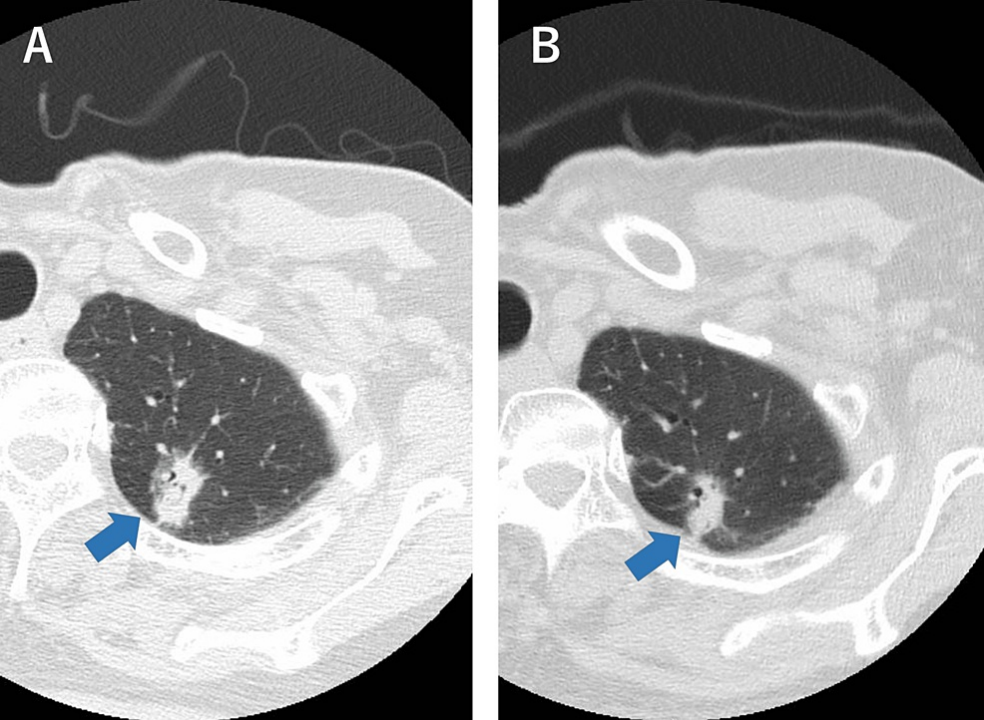
Chest computed tomography Computed tomography obtained (A) before and (B) three months after stereotactic ablative radiotherapy for lung cancer in the left upper lobe. Blue arrows indicate the lesion.

A lung lesion in the left upper lobe was found in an 89-year-old Japanese woman during a follow-up for breast cancer (Figure 1).

**Figure 2:**
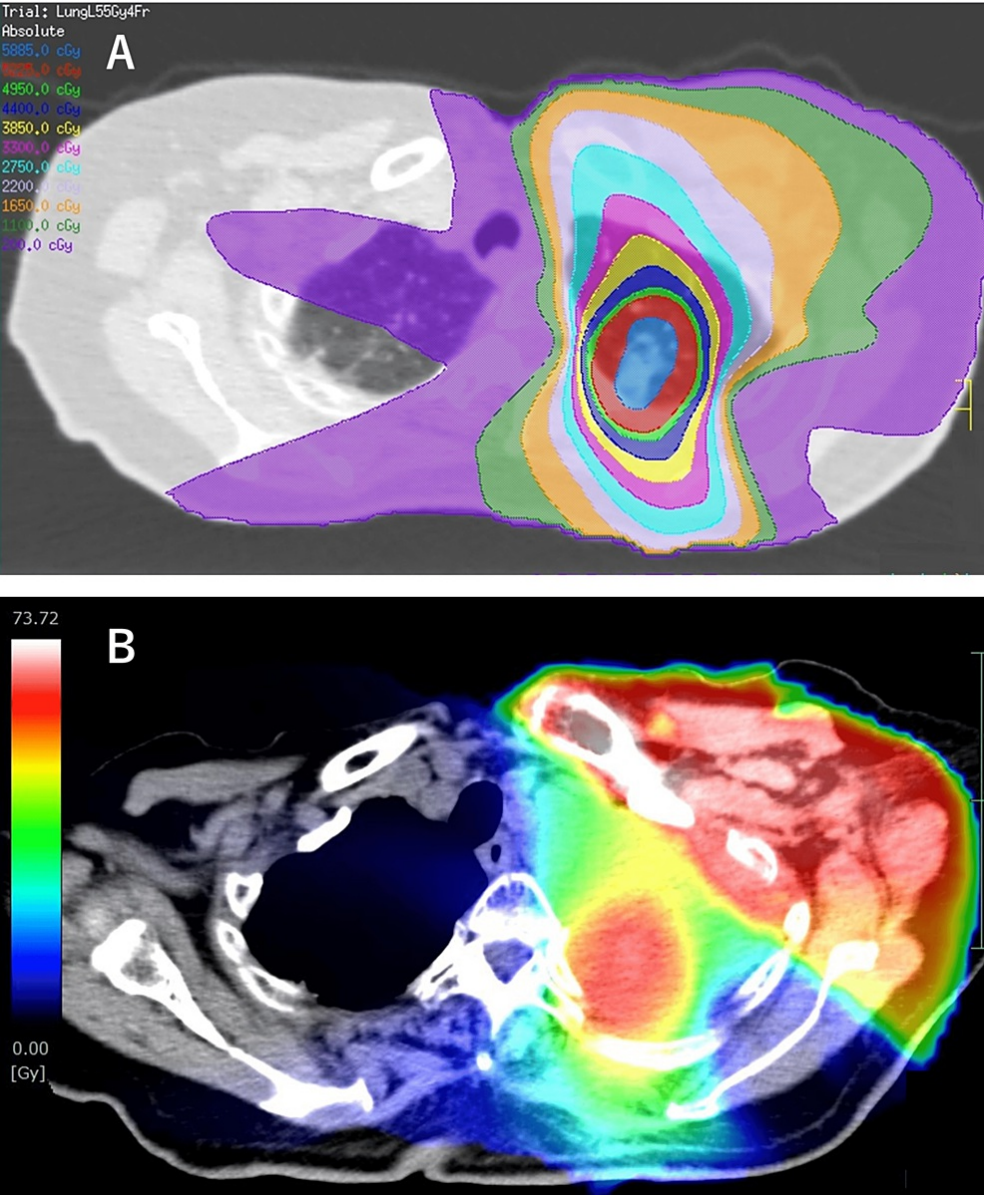
Treatment plan of stereotactic ablative radiotherapy for the left upper lobe lesion The treatment plan of stereotactic ablative radiotherapy for the left upper lobe lesion (A). The cumulative dose distribution of the previous two radiation treatment plans, consisting of breast irradiation 10 years prior and the current stereotactic ablative radiotherapy, was assessed by radiation therapy planning support software, SYNAPSE Radiotherapy provided by FUJIFILM Corporation (Tokyo, Japan).

She had a medical history of early-stage breast cancer (pT2N0M0) treated with partial resection and postoperative radiotherapy (50 gray (Gy) in 25 fractions) 10 years prior. The lung lesion was 1.3 cm in diameter and computed tomography (CT)-guided biopsy revealed adenocarcinoma with an epidermal growth factor receptor exon 21 L858R point mutation. The patient preferred a curative treatment strategy. The multidisciplinary team of our hospital recommended SABR to the patient rather than surgery, considering her age, and she chose SABR. SABR using 55 Gy in four fractions was administered to the lesion (Figure 2).

The gross tumor volume (GTV) was delineated by planning CT. No expansion was used in the GTV to create a clinical target volume (CTV). Internal target volume (ITV) was generated by combining CTV contours from all respiratory phases. The planning target volume (PTV) was defined as ITV plus an isotropic margin of 5 mm. The prescribed dose was planned to cover 95% of the PTV volume. The volume of the lungs receiving >20 Gy (V20) and >5 Gy (V5) was 6.38% and 9.19%, respectively, with a mean lung dose of 3.35 Gy.

**Figure 3:**
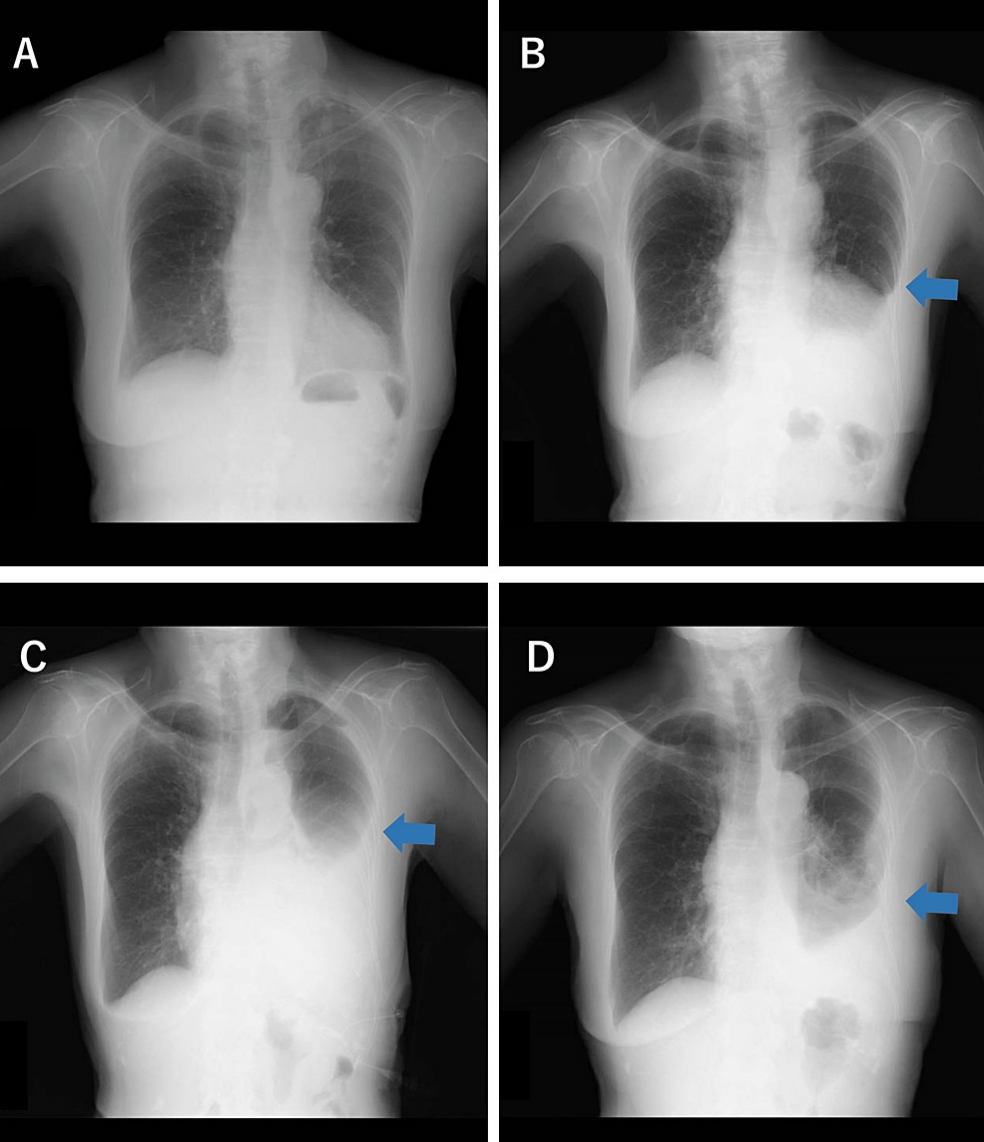
Chest X-ray Chest X-ray of the patient (A) before radiotherapy, (B) 18 months after radiotherapy, (C) 36 months after radiotherapy, and (D) 46 months after radiotherapy. Blue arrows indicate the pleural effusion.

Chest CT revealed mild shrinkage without evidence of recurrence at the three-month follow-up (Figure 1B). Six months after SABR treatment, a chest X-ray revealed left-sided pleural effusion. The pleural effusion gradually increased, and the patient experienced slight dyspnea and underwent thoracentesis 18 months after SABR treatment (Figure 3).

Pleural fluid analysis revealed exudative effusion with no evidence of bacteria in the fluid culture. The cytopathological analysis revealed no malignancy (Table 1).

**Table 1:** Pleural effusion analysis at several time points PF: pleural fluid; S: serum.

Variables	18 months	34 months	36 months	37 months
Total protein (PF) (g/dL)	4.0	4.7	4.4	4.6
Albumin (PF) (g/dL)	-	2.8	2.6	2.5
Lactate dehydrogenase (PF) (U/L)	122	135	142	116
Triglycerides (PF) (mg/dL)	12	16	-	-
Amylase (PF) (U/L)	13	15	13	15
Carcinoembryonic antigen (PF) (ng/mL)	4.1	-	-	-
Adenosine deaminase (PF) (U/L)	19.5	22.3	-	-
Hyaluronic acid (PF) (ng/mL)	4730	7100	-	-
Total protein (S) (g/dL)	6.6	-	6.7	-
Lactate dehydrogenase (S) (U/L)	205	-	265	205
Glucose (S) (mg/dL)	117	141	112	136
Bacterial culture (PF)	Negative	Negative	Negative	Negative
Cytology (PF)	Class 2	Class 1	Class 2	Class 2

She had no severe chronic heart disease and had intact renal and liver functions. Therefore, we considered that the pleural effusion originated from a radiation injury. After thoracentesis, temporary symptom relief was achieved; however, it gradually increased. At 34, 36, and 37 months after SABR treatment, the patient underwent repeated therapeutic thoracentesis for pleural fluid drainage (Figure 3C and Table 1). Pleural fluid analysis revealed no significantly different results from the first thoracentesis, and no malignant cells. A daily dose of 25 mg spironolactone and 20 mg furosemide was administered after the thoracotomy. Pleural effusion gradually decreased 46 months after SABR (Figure 3D). The treated lesion in the left upper lobe showed no evidence of recurrence. She had only slight dyspnea and no decrease in her level of activities of daily living.

## Discussion

This case report describes a rare but potentially serious complication of intractable pleural effusion after SABR for early-stage lung cancer. In the present case, the patient developed pleural effusion that was refractory to conservative management and required multiple interventions, including repeated thoracentesis. Zhao et al. previously investigated the risk factors for radiation-induced pleural effusion in patients with lung cancer receiving thoracic radiation therapy and found that whole-lung V5 was the most significant independent risk factor for symptomatic pleural effusion [6]. Harder et al. analyzed 335 patients who underwent SABR and investigated various pulmonary and cardiac dosimetric parameters for radiation pneumonitis [7]. They found that whole lung V10 was the strongest dosimetric predictor of grade ≥2 radiation pneumonitis, and mean lung dose was the most significant predictor of grade ≥3 radiation pneumonitis. These studies also highlighted the importance of monitoring and managing symptomatic pleural effusion in patients with lung cancer receiving radiotherapy.

Although the exact mechanism of pleural effusion after SABR is not fully understood, it is believed to be related to radiation-induced damage to the lymphatic vessels and microvasculature in the pleural space [8]. This can lead to increased vascular permeability and impaired fluid drainage, resulting in pleural fluid accumulation. In some cases, fluid accumulation can be severe and difficult to manage, leading to an intractable pleural effusion [9]. Although conservative measures such as thoracentesis and chest tube drainage can be effective in managing pleural effusion, as demonstrated in this case, some patients may require more invasive interventions such as surgical pleurodesis to achieve long-term symptom resolution [10].

Clinicians should be aware of the potential for intractable pleural effusion after SABR for lung cancer and closely monitor patients for this complication. Additionally, prompt intervention and consideration of more invasive measures, such as pleurodesis, may be necessary in cases of refractory pleural effusion. Further studies are needed to better understand the risk factors and mechanisms of pleural effusion after SABR and to optimize management strategies for this complication.

## Conclusions

In conclusion, this case report highlights a rare but serious complication of intractable pleural effusion after SABR for early-stage lung cancer. Previous studies have identified several significant risk factors for radiation-induced pleural effusion after SABR.

Clinicians should closely monitor patients for this complication and be prepared to intervene promptly if necessary. Further research is required to optimize management strategies and improve outcomes in patients with intractable pleural effusion after SABR.
